# Supplementary material for: Neurexophilin4 is a selectively expressed α-neurexin ligand that modulates specific cerebellar synapses and motor functions
Source: eLife. 2019 Sep 16;8:e46773. doi: 10.7554/eLife.46773 (PMC6763262; doi:10.7554/eLife.46773)
Supplement: Figure 1—source data 1. [file elife-46773-fig1-data1.docx]

**Figure 1-source data 1. Brain regions expressing *Nxph4.***

| Brain circuits | Specific regions |
| --- | --- |
| Olfactory bulb | Glomerular layer |
|  | Mitral cell |
|  | Accessory olfactory bulb |
| Cerebral cortex | Layer 6b |
| Sensory circumventricular organs + projections | Subfornical organ |
|  | Medial preoptic nucleus |
|  | Lateral hypothalamic area |
|  | Area postrema |
|  | Nucleus of vagus nerve |
| Mammillary body + projections | Mammillary bodies |
|  | Presubiculum |
|  | Dorsal tegmental nucleus |
| Cerebellar-related circuits | Golgi cells |
|  | Deep cerebellar nuclei |
|  | Medial/superior vestibular neucleus |
|  | Parasolitary nucleus |
|  | External cuneate nucleus |
|  | Pontine nuclei |
| Cochlear related circuits | Ventral cochlear nucleus |
|  | Inferior colliculus |
| Others | Amygdala (BLA, PLCo, PMCo, MePV) |
|  | Hypothalamus (DMD/DMV) |
|  | Piriform cortex |
|  | Zona inserta |
|  | EW region |
|  | Paranigral nucleus |
|  | Locus coeruleus |

BLA: basolateral amygdala

PLCo: posterolateral cortical amygdala

PMCo: posteromedial cortical amygdala

MePV: medial amygdala, posteroventral part

DMD: dorsal medial hypothalamus, dorsal part

DMV: dorsal medial hypothalamus, ventral part
